# Effect of Spatial Smoothing on Task fMRI ICA and Functional Connectivity

**DOI:** 10.3389/fnins.2018.00015

**Published:** 2018-02-02

**Authors:** Zikuan Chen, Vince Calhoun

**Affiliations:** ^1^The Mind Research Network and LBERI, Albuquerque, NM, United States; ^2^Department of Electrical and Computer Engineering, University of New Mexico, Albuquerque, NM, United States

**Keywords:** task fMRI, independent component analysis (ICA), task function mapping, function connectivity (FC), spatial smoothing, task correlation, spatial correlation (scorr), correlation scale invariance

## Abstract

Spatial smoothing is a widely used preprocessing step in functional magnetic resonance imaging (fMRI) data analysis. In this work, we report on the spatial smoothing effect on task-evoked fMRI brain functional mapping and functional connectivity. Initially, we decomposed the task fMRI data into a collection of components or networks by independent component analysis (ICA). The designed task paradigm helps identify task-modulated ICA components (highly correlated with the task stimuli). For the ICA-extracted primary task component, we then measured the task activation volume at the task response foci. We used the task timecourse (designed) as a reference to order the ICA components according to the task correlations of the ICA timecourses. With the re-ordered ICA components, we calculated the inter-component function connectivity (FC) matrix (correlations among the ICA timecourses). By repeating the spatial smoothing of fMRI data with a Gaussian smoothing kernel with a full width at half maximum (FWHM) of {1, 3, 6, 9, 12, 15, 20, 25, 30, 35} mm, we measured the spatial smoothing effects. Our results show spatial smoothing reveals the following effects: (1) It decreases the task extraction performance of single-subject ICA more than that of multi-subject ICA; (2) It increases the task volume of multi-subject ICA more than that of single-subject ICA; (3) It strengthens the functional connectivity of single-subject ICA more than that of multi-subject ICA; and (4) It impacts the positive-negative imbalance of single-subject ICA more than that of multi-subject ICA. Our experimental results suggest a 2~3 voxel FWHM spatial smoothing for single-subject ICA in achieving an optimal balance of functional connectivity, and a wide range (2~5 voxels) of FWHM for multi-subject ICA.

## Introduction

A functional magnetic resonance imaging (fMRI) experiment captures brain activity via a time series of images or a spatiotemporal series (represented by a 4D dataset). For a task fMRI study, where the task paradigm was predefined (designed), we can extract the task activation map through the use of task response correlation (voxelwise temporal correlations against the task stimuli Moritz et al., 2000; Chen and Calhoun, 2015, 2016) or independent component analysis (ICA) (Calhoun et al., 2001; Calhoun and Adali, 2012; Chen and Calhoun, 2016). Prior to ICA, the raw fMRI data were usually subject to a standard SPM (http://www.fil.ion.ucl.ac.uk/spm/software/spm8/) preprocessing pipeline, including motion correction, spatial normalization, and spatial smoothing (filtering). Here, we report the effect of spatial smoothing on the ICA-based brain task function analysis through a variety of spatial smoothing settings during fMRI data preprocessing.

A spatiotemporal fMRI dataset acquired from a brain fMRI scan represents a spatiotemporal dynamic evolution of brain activity, which can be decomposed into subactivities through an ICA approach (Calhoun and Adali, 2012; Duff et al., 2012; Chen and Glover, 2015). The ICA output consists of a collection of maximally independent components, with each ICA component representing a brain subactivity or a coherent network in a pair of spatial mode (ICA map in variable **r**) and temporal mode (ICA timecourse in variable t). The functional network in an ICA component map may contain one or more local activation blobs in brain space, which fluctuate similarly as described by the ICA timecourse (intra-network coherence). By calculating the temporal correlations among ICA timecourses, we obtain a function network connectivity (FC) matrix (Jafri et al., 2008; Chen et al., 2017a). The pattern in an FC matrix is determined by the ordering (or labeling) of the ICA timecourses. For a task fMRI ICA study, we can use the predefined task paradigm (a cue of timecourse) to order the ICA components in an order of task correlation, such that we may compare ICA-based FC matrices generated from different ICA outputs (where the ICA components are always disordered). Another important reason to work on task fMRI ICA is that the ICA technique can successfully extract the primary task performance component (Duff et al., 2012; Xu et al., 2013; Chen and Calhoun, 2016), which provides a useful reference point for conducting analysis.

Spatial smoothing is a typical preprocessing step for fMRI analysis, which is usually implemented through a Gaussian kernel with a specification of full width at half maximum (FWHM) (Friston et al., 1995, 2000; Lowe and Sorenson, 1997; Liu et al., 2017). Its primary goal is to suppress spatial noise and enhance the signal to noise ratio (SNR). Recent research (Liu et al., 2017) shows that the spatial smoothing (with a large smoothing kernel) may cause a correlation-based functional overestimation that can be explained with a correlation scale invariance theory. Since the FC calculation consists of temporal correlation, this may also be impacted by the spatial smoothing effect as dictated by the correlation scale invariance theory (Liu et al., 2017). With this logic, we may partially explain the spatial smoothing effect on the task-ordered FC matrix analysis.

The fMRI signals are noisy and weak (accounting for less than a 5% change in energy consumption Raichle, 2010; Gonzalez-Castillo and Bandettini, 2017). This can be mitigated by working on multi-subject data; for example, through a group ICA (Calhoun et al., 2001; Esposito et al., 2005). Accordingly, we carried out our study on a multi-subject experiment (20 subjects). Notwithstanding, there emerges a research trend on individual fMRI study under a claim that “the data from a single subject are actually meaningful and reliable” (Finn et al., 2015; Vogt, 2015). In support of this advocacy, we also provided a study on a single-subject experiment in which we demonstrated our data analysis method in technical details along with a comparison to the multi-subject experiment.

## Methods

### fMRI data from subject experiments

The one single-subject fMRI dataset, which was originally acquired for an inverse fMRI study (Chen et al., 2013; Chen and Calhoun, 2015), was reused herein for demonstrating the task-evoked ICA technique (reported below). This single-subject dataset was acquired by scanning one heathy subject (age 44, male) in a Siemens TrioTim 3T scanner while performing a finger tapping task. This study used the following experimental settings: standard GRE-EPI sequence, TR/TE = 3000/29 ms, flip angle = 75°, no slice oblique, voxel size = 3 × 3 × 3 mm^3^, matrix = 64 × 64 × 32, and 165 timepoints (five repetitions of [15 OFF, 15 ON] plus 15 OFF). The fMRI data acquisition by spatial sampling with [3,3,3] mm intervals produced a timeseries of isotropic images.

We also acquired a group of task fMRI datasets from 20 subjects (healthy, age 38 ± 10, 13 males) using the same 3T scanner at the Mind Research Network (MRN) and similar scan parameters (except for slice oblique = 20° and voxel size = 3.75 × 3.75 × 4.55 mm^3^). We designate the fMRI data acquired by spatial sampling with [3.75,3.75,4.55] mm intervals as “anisotropic.” This subject group did not include the subject who was used for isotropic fMRI data as aforementioned.

All of the fMRI data were acquired by scanning adult volunteers at MRN. The MRI scans were approved by the Institutional Review Board at MRN. Written consent was obtained from each subject before scanning. We used one isotropic dataset and a group of 20 anisotropic datasets for a comparative study on the effect of spatial smoothing on single-subject and multi-subject ICA data analyses.

### Data preprocessing

A timeseries of fMRI data (a 4D dataset) was subject to standard SPM preprocessing, including motion correction (timeseries image alignment), spatial normalization (resampled into voxels of 3 × 3 × 3 mm^3^ in MNI brain space), and spatial smoothing with a Gaussian smoothing kernel with a FWHM. In order to observe the effect of spatial smoothing, the spatial smoothing procedure was repeated with a range of FWHM = {1, 3, 6, 9, 12, 15, 20, 25, 30, 35} mm.

### Independent component analysis (ICA)

Upon completion of the SPM preprocessing, we performed a spatial ICA to generate a collection of ICA components (consisting of spatial maps and timecourses in pairs). Let X[**r**, t] denote a 2D matrix as generated by an arrangement of space (**r**) × time (t) of the 4D spatiotemporal data; the group ICA decomposition is represented by Chen et al. (2017a)

(1) $$\begin{array}{l} X\left[\text{r},t\right]=\overset{N}{\underset{n=1}{\sum }}X_{n}^{ICA}\left[\text{r}\right]⊗X_{n}^{ICA}\left[t\right] \end{array}$$

where ⊗ is a product operator (signifying space-time separation), N the component number (empirically specified), and {$X_{n}^{ICA}\left[\text{r}\right],X_{n}^{ICA}\left[t\right]$} represents the pair of spatial map and timecourse of the n*th* ICA component.

In a similar way, we applied a group-level ICA to the multi-subject data by stacking the 2D space × time matrix of individual subject data into an augmented 2D matrix along the time dimension. In the results of group ICA [in a similar formulation in Equation (1)], we obtained a collection of *N* pairs of aggregate ICA components in which an aggregate ICA spatial map represents a common subactivity, and the associated timecourse represents its temporal evolution.

Upon ICA decomposition of task fMRI data, we conducted the following task function analyses: the task identification in an ICA component, the FC among the ICA components, and the spatial overlap among the ICA components.

### Task identification from ICA component

We let task[t] denote the designed task paradigm (stimuli timecourse); we can then find the ICA-extracted task activation, denoted by a pair {$X_{task}^{ICA}\left[\text{r}\right],X_{task}^{ICA}\left[t\right]$}, which are determined at the maximal task correlation by

(2)         XtaskICA[r]=Xn*ICA[r],XtaskICA[t]=Xn*ICA[t]       with n*=arg maxn{corr(XnICA[t],task*[t])and task*[t]=conv(task[t],hrf[t])

where *corr* denotes a Pearson correlation (correlation coefficient), *conv* is a convolution, and *argmax* finds the argument at the maximum condition. The designed square waveform task[t] was convolved with a canonical hemodynamic function (*hrf* [t], available in SPM package) to account for the sluggish and up-shooting BOLD response.

**Figure 1:**
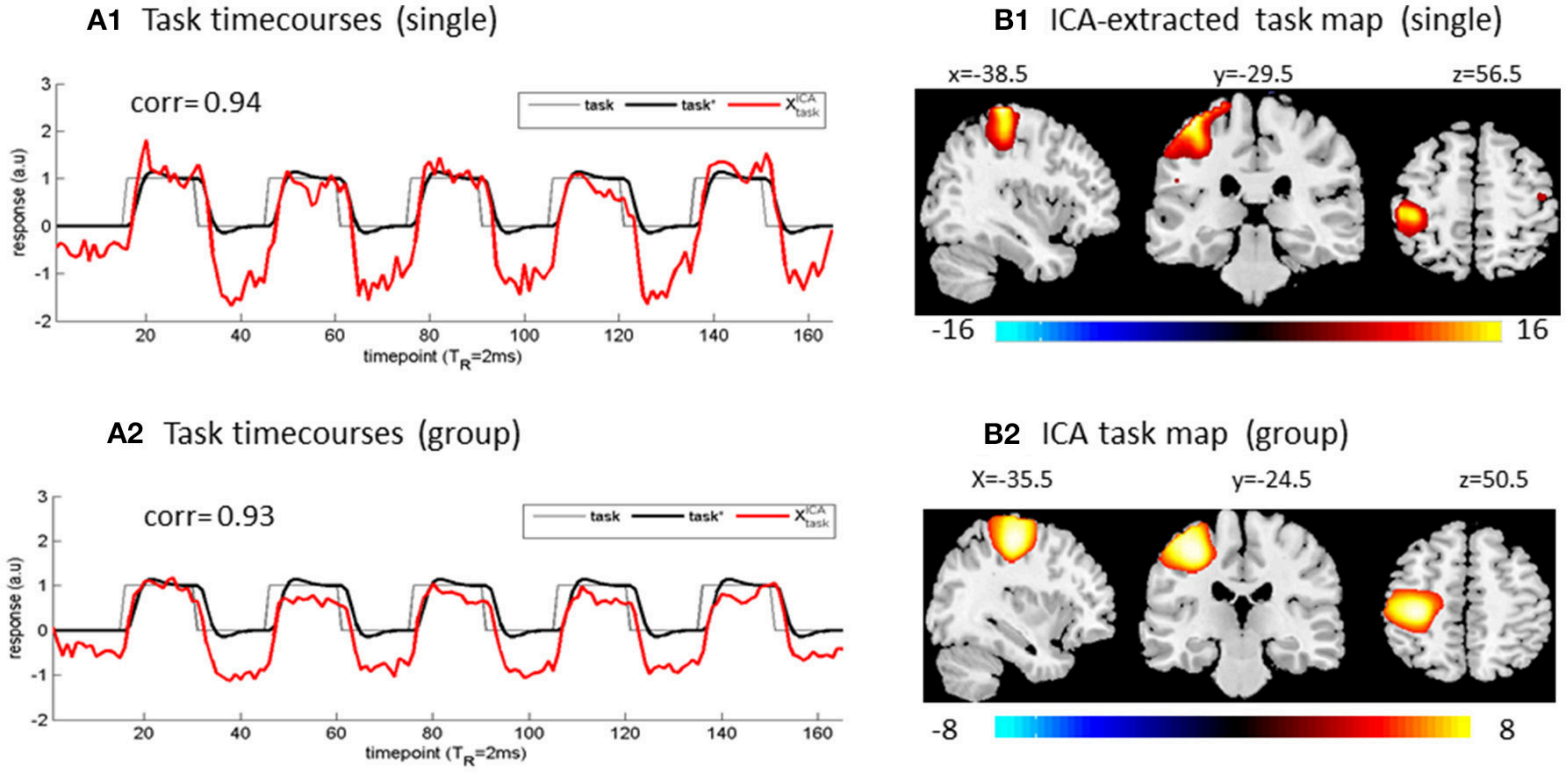
ICA-based task extractions from **(A1,B1)** a single-subject dataset and **(A2,B2)** group of 20 subject datasets. The designed task paradigm was a timecourse of square waveform. The (*x*,*y*,*z*) coordinates denote activation foci in MNI brain space.

In Figure 1, we demonstrate the task activation extractions from our finger tapping experiment data (Figures 1A1,B1) for single-subject ICA, and (Figures 1A2,B2) for multi-subject ICA). To a great extent, a high maximal correlation (e.g., *corr* (${\text{X}}_{\text{n}}^{*}$[*t*], task^*^[*t*]) > 0.9) serves as a justification (criterion) for ICA-based brain function analysis (Chen and Calhoun, 2016). In practice, the success of ICA-based task function extraction can always be verified with a spatial conformation of an ICA spatial map using the well-established brain function atlas ($X_{task}^{ICA}\left[\text{r}\right]$ verification) and a reproduction of the task paradigm in an ICA component ($X_{task}^{ICA}\left[t\right]$ verification).

### Task-correlation ordering of ICA components

Using the temporal correlation of the task ICA timecourse against all other ICA timecourses, we may relabel the ICA components in a descending order as given by

(3) $$\begin{array}{l} n_{1}>n_{2}\;s.t.\text{|}taskcorr\text{(}n_{\text{1}}\text{)| > |}taskcorr\text{(}n_{\text{2}}\text{)|}\\ \text{with }taskcorr\text{(}n\text{) =}corr\left(X_{n}^{ICA}\left[t\right],X_{task}^{ICA}\left[t\right]\right) \end{array}$$

where s.t. stands for “such that” or “subject to.” The new ICA index labeling in Equation (3) gives rise to $X_{task}^{ICA}\left[t\right]$ = $X_{1}^{ICA}\left[t\right]$ (n^*^ = 1 as such). The first few ICA components, which assume high |*taskcorr*| values (e.g., > 0.9), may be considered as high task-relevant components; whereas the last ICA components, which assume small |*taskcorr*| values (close to 0), are interpreted as task-irrelevant components representing brain autonomous subactivities not related to the task performance.

**Figure 2:**
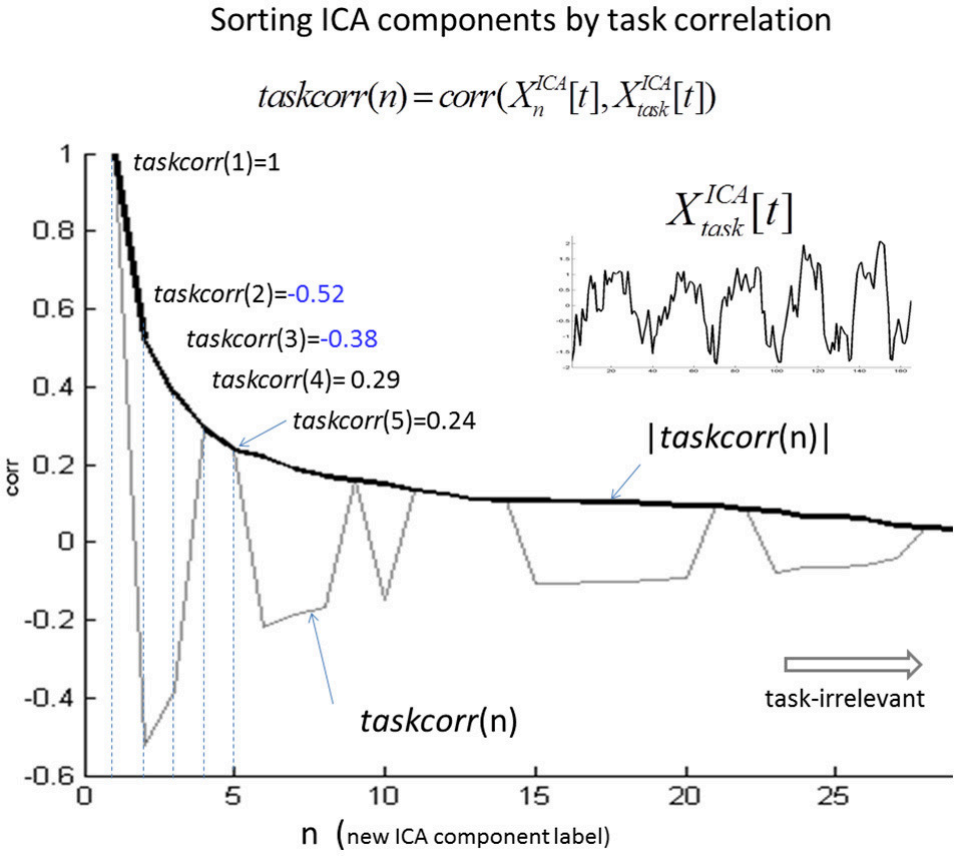
Illustration of ICA component relabeling according to a descending order of task correlation (|*taskcorr*|). At the head of the ordering are the task-relevant components (e.g., |*taskcorr|* > 0.8) and at the end are the task-irrelevant components (e.g., |*taskcorr|* < 0.1).

In Figure 2, we illustrate the ICA component relabeling in a descending order of |*taskcorr*| (defined in Equation 3) from a typical single-subject ICA study (spatial smoothing with a 2-voxel (6 mm) FWHM). In Supplementary Figure S1, we show the first nine components (|*taskcorr*| > 0.15) from this single-subject ICA decomposition.

Since ICA is a stochastic algorithm, its output components may be somewhat different for various runs; we used the ICASSO algorithm (Himberg et al., 2004) (implemented in the GIFT software (http://mialab.mrn.org/software/gift) by running ICA five times and selecting the best run (Ma et al., 2011). In order to compare the ICA components generated from various runs with different spatial smoothing, we used the group-information-guided (GIG) back-reconstruction to produce the same number of ICA components in a fixed component arrangement from different runs. The resulting ICA components are then ordered according to |*taskcorr*| in Equation (3), thus facilitating the FC matrix comparison (see later).

### Task activation volume measurement

Upon establishing the task pattern recognition in an ICA component (label n^*^ in Equation 3), we can measure the primary task activation volume at the activation foci (centroid of the activation blob). In the ICA output, the ICA-extracted primary task activation map $X_{task}^{ICA}\left[\text{r}\right]$ is represented in the z-score of a statistical t-test map. We can use this to calculate the activation region volume by counting the voxels around the task foci that assume values larger than a threshold (empirically determined) (Chen et al., 2017b). When we let *taskV* denote the task activation volume at a threshold *th*, it is measured by

(4) $$\begin{array}{l} \;taskV=\sum_{r\in \Omega (r_{0})}step(X_{task}^{ICA}[r]>\text{th})\;(\text{voxel})\\ \text{with }step(x)\;=\{\begin{array}{cc} 1, & x>0\\ 0, & \text{else} \end{array} \end{array}$$

where Ω(**r**_0_) denotes a local spatial region around **r**_0_ as visually determined at the task activation foci. The task activation volume (*taskV*) is represented by a number of voxels, which can be converted to a spatial volume in units of cm^3^ (e.g., 1 voxel = 0.3 × 0.3 × 0.3 = 0.027 cm^3^ for isotropic 3-mm voxels).

### Task-ordered FC matrix

With the new ICA component labeling (in Equation 3), we may establish a FC matrix, as given by

(5) $$\begin{array}{l} FC\left[n_{1},n_{2}\right]=corr\left(X_{n_{1}}^{ICA}\left[t\right],X_{n_{2}}^{ICA}\left[t\right]\right). \end{array}$$

The FC matrix is bound in a value range [−1, 1], in dimensionless unit. A large FC value signifies a high correlation between two networks (components). A positive and negative sign represents a para-correlation and anti-correlation, respectively. It is noted that the FC matrix contains *taskcorr* in the 1st row (or column).

An FC matrix is a representation of temporal coupling (synchrony). An on-diagonal block represents a subgroup of correlations (a functional clique) that behave with a high synchrony (coherence), and an off-diagonal block indicates similar correlations between two functional subgroups. A block in an FC matrix indicates the local homogeneity among the function couplings. An average (*mean* value) of an FC matrix may be due to either the balance of positive and negative correlations (cancellation of prominent positive and negative correlations) or due to no correlations (small correlations *per se*). Thus, we can differentiate these two scenarios using the standard deviation (*std*) of the FC matrix (Chen et al., 2017b). A large positive swing cancels out a large negative swing in the *mean* value of an FC matrix, but the large positive and negative swings are reflected in the *std* value. That is, a high *std* value of an FC matrix indicates a balance of strong positive and negative couplings, while a small *std* value indicates irrelevance or no coupling. Therefore, we suggest the use of *mean*(FC) ± *std*(FC) for numerical characterization of the FC matrices generated from different spatial smoothing.

### Task-ordered inter-component spatial correlation (*scorr*) matrix

In a similar way to the temporal correlation matrix in Equation (5), we calculate the spatial correlation (*scorr*) matrix among the ICA spatial components by

(6) $$\begin{array}{l} scorr\left[n_{1},n_{2}\right]=corr\left(X_{n_{1}}^{ICA}\left[\text{r}\right],X_{n_{2}}^{ICA}\left[\text{r}\right]\right) \end{array}$$

Obviously, a small *scorr* value represents a small spatial overlap between the correlation patterns. Again, we may also characterize the spatial smoothing effect on the spatial overlap of ICA components in terms of *mean*(*scorr*) ± *std*(*scorr*).

### Correlation scale invariance

According to the definition of the Pearson correlation coefficient (Gonzalez and Wood, 2008), the correlation scale invariance property (Hoeffding, 1994) is expressed by Liu et al. (2017):

(7) $$\begin{array}{l} corr\left(a_{1}s_{1},a_{2}s_{2}\right)=corr\left(s_{1},s_{2}\right)\text{ for }a_{1}\neq 0\text{ and }a_{2}\neq 0. \end{array}$$

where s_1_ and s_2_ represent two signals, and *a*_1_ and *a*_2_ are two arbitrary scales. Note that the *corr* value is invariant to the arbitrary scaling of the signals. For instance, a strong response signal s_1_ (with a scale a_1_ = 1) and a weak response signal 10^−3^s_1_ (with a scale of *a*_1_ = 0.001) produce the same correlation value with the stimuli (s_2_) as a result of *corr*(s_1_, s_2_) = *corr*(10^−3^s_1_, s_2_). In extreme noiselessness, the correlation scale invariance leads to a complete failure of the correlation-based functional mapping (Liu et al., 2017). It is the ubiquitous noise that shapes a correlation map according to noise level and signal strength. Clearly, a smoothing may change the noise level, which in turn influences the correlation map. Since the functional connectivity (FC in Equation 5) and the corresponding functional spatial overlap (*scorr* in Equation 6) are defined by a *corr* value, both of them may be affected by spatial smoothing in certain circumstances.

## Results

We report here our findings from the two experiments: the single-subject experiment as designated by “single,” and the multi-subject experiment with the “group” designation. During SPM preprocessing, we performed spatial smoothing with a Gaussian kernel with a kernel size as specified in terms of FWHM (in units of mm). By repeating the spatial smoothing procedure for a range of FWHM = {1,3,6,9,12,15,20,25,30,35} mm while carrying out the other routines, we studied the effect of spatial smoothing effects.

### Task identification from ICA components

We applied ICA to the single-subject dataset and generated 40 ICA components [*N* = 40 in Equation (1), empirically specified for reliable primary task activation extraction in a moderate brain function decomposition]. First, we show that ICA can reliably extract the task activation mode as determined by the maximal correlation criterion in Equation (2). In Supplementary Figures S2, S3), we show the ICA-extracted task paradigm timecourses as obtained across a span of spatial smoothing settings (designed with different FWHMs) for single-subject ICA (Supplementary Figure S2) and multi-subject ICA (Supplementary Figure S3). All of the ICA-extracted task timecourses have a high correlation with the predefined task design timecourses (*corr* > 0.9). Correspondingly, we show the ICA-extracted task activation maps for the finger tapping performance in Supplementary Figures S4, S5. It is clear that the ICA-extracted task activation patterns are consistently reproduced at the motor cortex after different spatial smoothing (the activation blobs were displayed with orthogonal slices at the same foci, as designed with the same (x,y,z) MNI coordinates).

**Figure 3:**
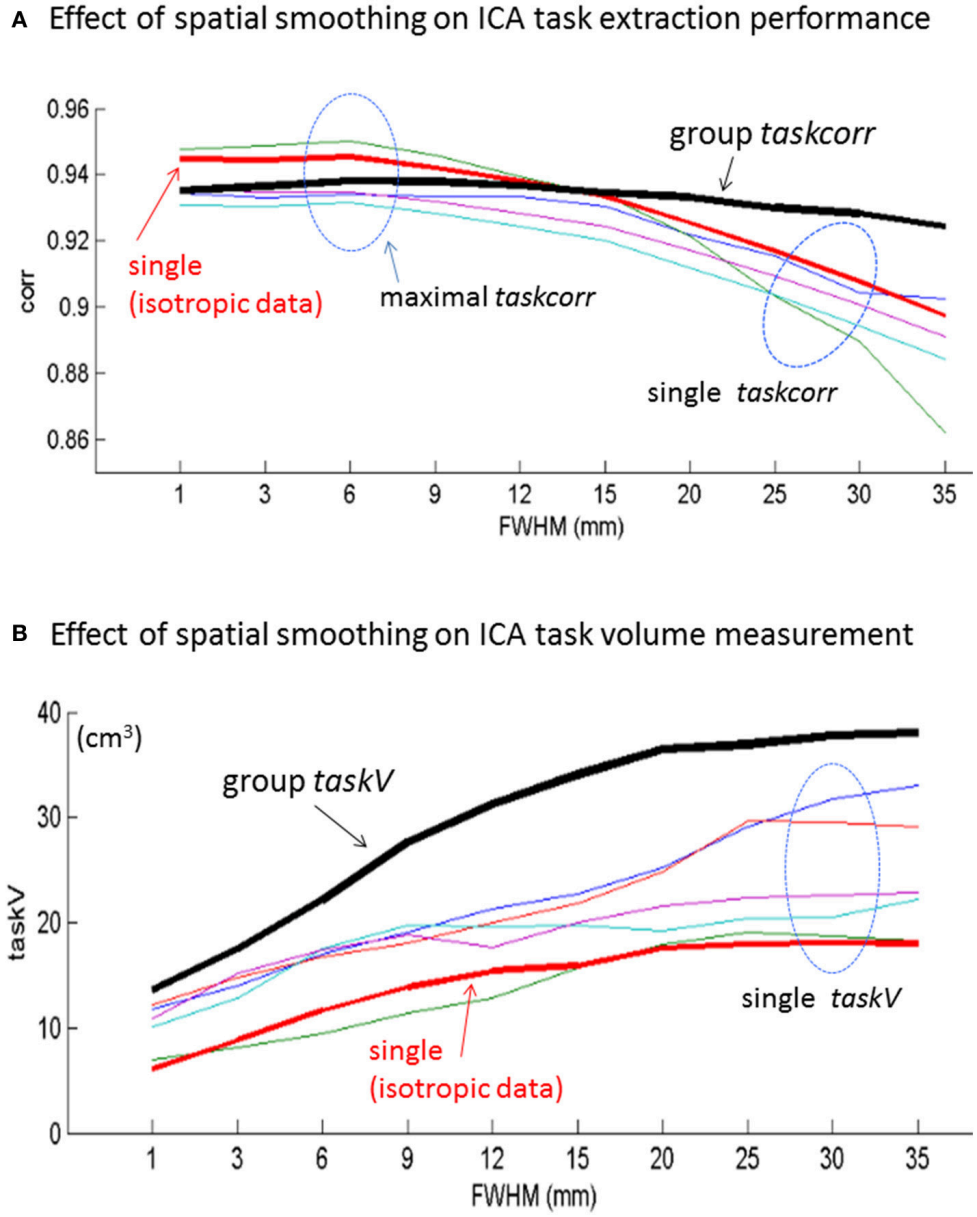
Effect of spatial smoothing of fMRI on **(A)** task extraction performances (in terms of maximal task correlation) and **(B)** task activation volume measurements for single-subject (one isotropic dataset and several anisotropic datasets) and multi-subject experiments.

With the ICA-extracted task component timecourse [determined by Equation (2)], we then quantified the ICA's task extraction performance in terms of maximal *taskcorr* (in Equation 3). With the ICA-extracted task component map, we calculated the task activation volume in terms of *taskV* (in Equation 4 with a threshold of z-score = 4). In Figure 3, we show the maximal task correlations and *taskV* measurements, for both the single-subject and multi-subject ICAs, with respect to spatial smoothing. In Figure 3A, we show that the task extraction performance of single-subject ICA decreases as smoothing FWHM increases, whereas that of multi-subject ICA increases slightly. Note that we display the group ICA *taskcorr* in a black thick plot; the one special single-subject *taskcorr* in a red thick plot (an isotropic dataset as acquired in 3 × 3 × 3 mm^3^ voxels); and together with several single-subject results from the group (no particular selection from the anisotropic datasets). Overall, the spatial smoothing reduces the ICA-based task identification performance of single-subject ICA more than that of group-subject ICA.

In Figure 3B, we show that both single-subject and multi-subject ICA decompositions yield an everlasting increase in *taskV* as the spatial smoothing FWHM increases. The spatial smoothing imposes more effect on multi-subject *taskV* measurement than on the single-subject *taskV* measurement. Specifically, the group-level *taskV* is higher above all the single-subject *taskV*, and the ever increasing *taskV* tends to saturate when FWHM > 20 mm (~6 voxels).

### Spatial smoothing effect on FC matrices

With a group information template generated by an ICASSO ICA on the 9 mm-FWHM-smoothed fMRI data, we obtained ICA decompositions (through GIG-ICA) from the spatially smoothed fMRI data with different FWHMs ({1, 3, 6, 9, 12, 15, 20, 25, 30,35} mm).

**Figure 4:**
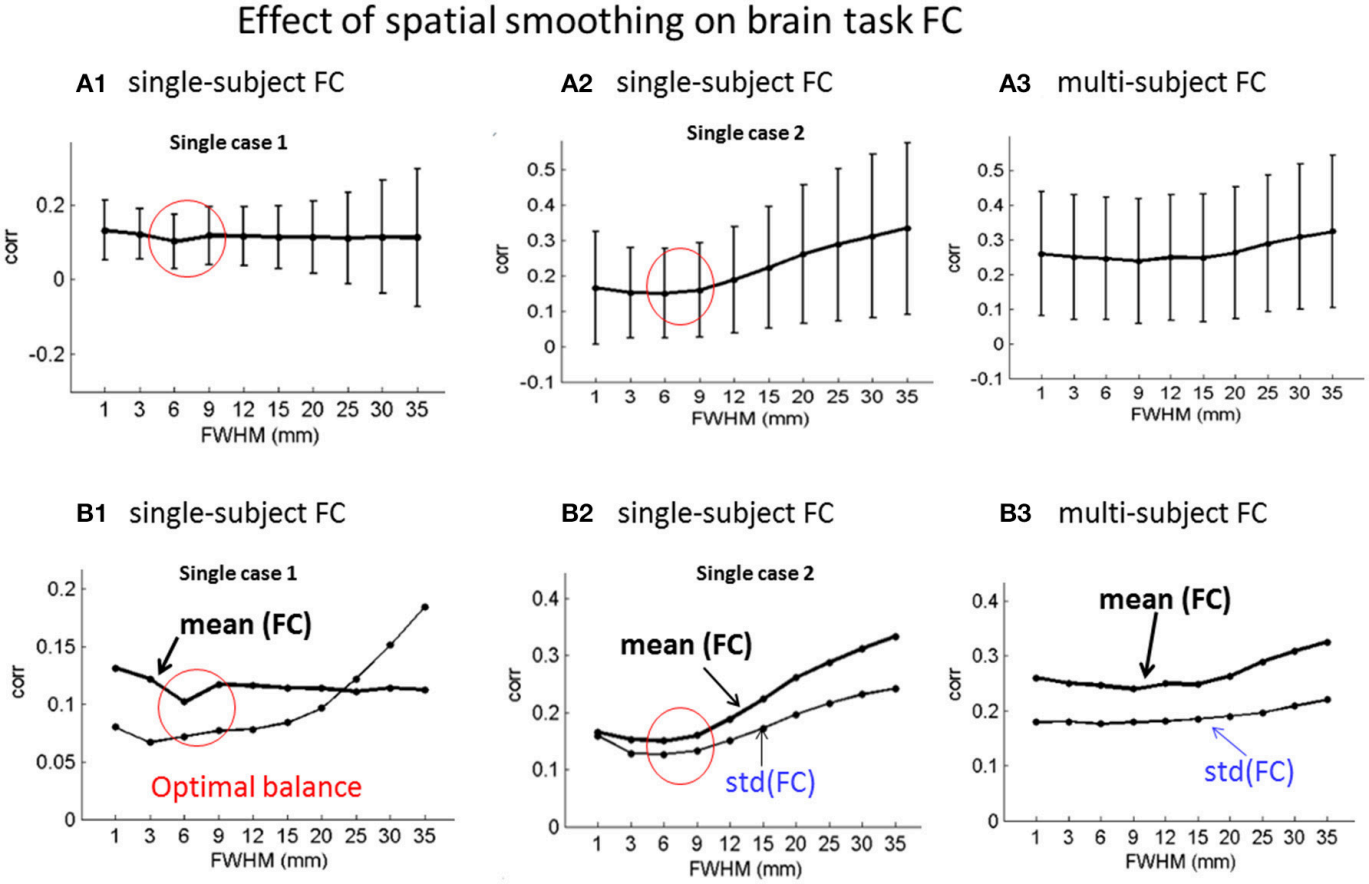
Effect of spatial smoothing on brain task ICA-based FC analyses in terms of *mean* (FC) ± *std*(FC)for **(A1,B1; A2,B2)** two exemplary single-subject ICA and **(A3,B3)** the 20-subject group ICA FC matrices under spatial smoothing in a range of FWHMs. **(A1–A3)** errorbar plots of *mean*(FC) ± *std*(FC); and **(B1–B3)** plots of *mean*(FC) and *std*(FC).

Based on ICA decomposition and ICA component relabeling (in a descending order of |*taskcorr*| in Equation 3), we calculated the FC in Equation (5). We show the FC matrices under different smoothing (specified by FWHM in units of millimeter) in Supplementary Figures S6, S7. The FC matrices generated from the ICA-decomposed components under the same smoothing are presented in Figure 4, in which we show two single-subject ICA cases in Figures 4A1,B1; A2,B2 and the multi-subject experiment in Figures 4A3,B3. It is seen that the coupling strength [in terms of *std*(FC)] increases for large-FWHM spatial smoothing for the single-subject ICA. In comparison, the spatial smoothing has less effect on the FC matrices of multi-subject ICA. The single-subject experimental results in Figures 4A1,B1; A2,B2 show that the optimal FC balance [in terms of minimal *mean*(FC)] reaches at the spatial smoothing with 2~3-voxel FWHM (corresponding to 6~9 mm). In comparison, the spatial smoothing has less effect on the multi-subject experiment (in Figures 4A3,B3).

### Spatial smoothing effect on *Scorr* matrices

We also calculated the spatial correlations among the ICA spatial components, as represented in *scorr* matrices (in Equation 6), with the same ICA component labeling as used for FC matrix calculation. The *scorr* matrices for the single- and multi-subject experiments are shown in Supplementary Figures S8, S9, respectively. It is notable that the *scorr* matrices assume small values (*corr* < 0.1), implying small spatial overlap of the ICA-decomposed components under different smoothing parameters.

**Figure 5:**
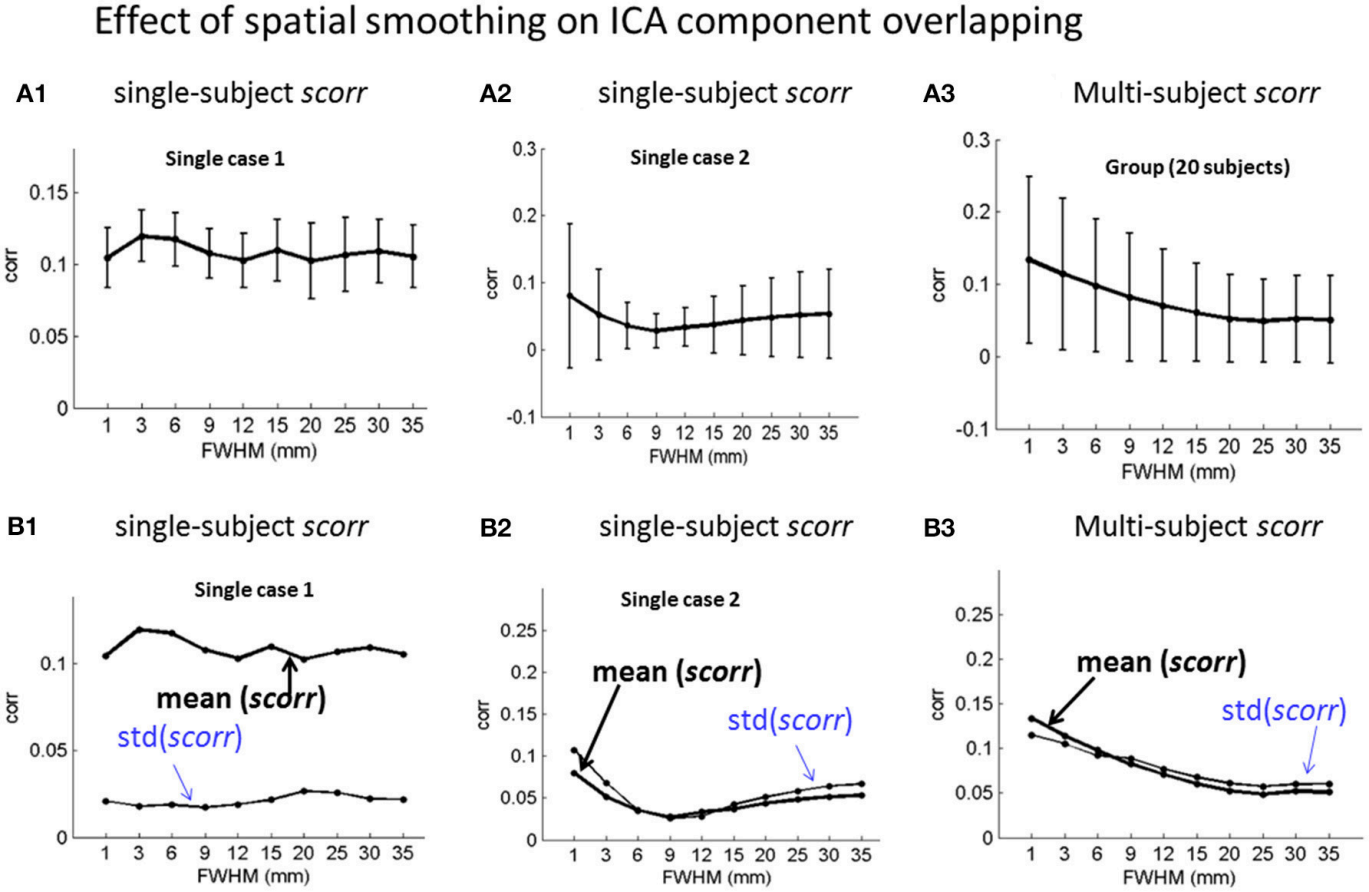
Effect of spatial smoothing on the spatial overlap of ICA-decomposed components with numerical characterization of *mean*(*scorr*) ± *std*(*scorr)* for **(A1,B1; A2,B2)** two exemplary single-subject ICA and **(A3,B3)** the 20-subject group ICA. **(A1–A3)** errorbar plots for *mean*(scorr) ± *std*(scorr); **(B1–B3)** the plots *mean*(*scorr*) and *std*(*scorr*).

Based on the *scorr* matrices, we calculated the *mean* ± *std* values and presented the errorbar plots in Figure 5 to show the spatial smoothing effect with two single-subject results (Figures 5A1,B1; A2,B2) and the group analysis result (Figures 5A3,B3). Bascially, our experimental data analyses results in Figure 5 show that the spatial smoothing has no obvious effects on the ICA component overlap because the overall *scorr* values are generally very small (*corr* < 0.2, see display scales).

## Discussion

For brain function analysis using fMRI data, spatial smoothing is always applied to the raw data for the sake of SNR gain. In practice, the spatial smoothing was usually implemented through the use of a Gaussian kernel with a certain FWHM. By applying spatial smoothing with a large span of FWHMs, we observed the effect of spatial smoothing on brain function analysis. In this paper, we report on spatial smoothing effects on ICA-based task fMRI data analysis in the context of task extraction capability (Figure 3A), task activation volume measurement (Figure 3B), functional connectivity (Figure 4), and spatial overlap among ICA-decomposed subactivities (Figure 5).

The reasons for us to use the task fMRI data, rather than resting-state data, to study the spatial smoothing effect are 2-fold. First, the designed task paradigm (predefined truth of task timecourse) allows us to justify the ICA-based brain function analysis as long as the task timecourse can be highly reproduced in an ICA component timecourse (e.g. with a maximum correlation > 0.9 in Figure 3A). Second, the predefined task timecourse can be used as a reference to sort the ICA components (e.g., in a descending order of |*taskcorr*|). In the task-correlation ordering, the most task-relevant components are gathered at the head of the list with high task correlations (in particular, the 1st one is the ICA-extracted primary task component), and the task-irrelevant components (such as noise and motion components) are sorted at the end of the list with small task correlations. This aspect is not available with resting-state fMRI data. Through the use of an ICASSO algorithm for reliable ICA decomposition (Himberg et al., 2004), we obtained a group information template for the subsequent GIG-ICA (Du and Fan, 2013; Du et al., 2016), thus facilitating the comparison among ICA components generated in different runs.

In our experimental demonstration, we provided one isotropic dataset and 20 anisotropic datasets, which all were acquired from different subjects performing the same finger tapping task inside the same scanner (3T Siemens TrioTim scanner at MRN). The MRI scanning used a standard GRE-EPI sequence with slight parameter differences in voxel size and slice oblique (see Methods). The single-subject datasets were then processed separately through the same ICA procedure. Our experimental data analyses show that the particular isotropic single-subject dataset produced the similar ICA task function analysis performance as did the several non-isotropic single-subject datasets under a range of spatial smoothing (see Figures 4, 5). Specifically, the spatial smoothing causes a reduction in task activation extraction performance (in terms of *taskcorr*) and an increase in task volume measurement (in terms of *taskV*), very much in a similar behavior. In comparison in Figures 4, 5, the spatial smoothing imposes a less effect on the group-level ICA (20 non-isotropic subject datasets.

With minimal error from SPM spatial resampling and spatial rotation, the particular single-subject isotropic data analysis provides a good representative of single-subject ICA. In terms of maximal |*taskcorr|* for task extraction performance, our experiments in Figure 3A suggest a 2~3-voxel FWHM for single-subject data spatial smoothing, which is roughly in agreement with the optimal empirical spatial smoothing setting (2-voxel FWHM) as previously reported (Pajula and Tohka, 2014; Chen et al., 2017b). In comparison, the group ICA task extraction performance is insensitive to the spatial smoothing, as evidenced in the flat *taskcorr* plot in Figure 3. In this particular experiment, we see that the optimal group ICA performance can be achieved through the use of a wide range of FWHM with roughly 2~5 voxels (corresponding to 6~15 mm). On the other hand, from Figure 3B, we observed that the group *taskV* tends to saturate beyond 6-voxel FWHM, suggesting no more than 6-voxel FWHM spatial smoothing for group-level ICA study.

Brain fMRI research has been widely performing at a group level, perhaps due to the weakness of brain fMRI signal (brain function response <5% baseline signal), but there is an emergence of fMRI analysis shifting to individual study (Chen and Calhoun, 2015, 2016; Finn et al., 2015; Vogt, 2015). In this work, we present a single-subject experiment to support the advocacy of individual fMRI study. The results indicate that the ICA is a powerful task fMRI analysis approach based on the success in task activation extraction (with *corr* > 0.90 in Figure 3A). In general, we observed similar effects of spatial smoothing on both the single-subject and multi-subject experiment data analyses (in despite of data acquisition with somewhat different experimental settings). In particular, we found that the spatial smoothing enhances the functional connection strength more on the single-subject than on the multi-subject data analyses (Figure 4).

The FC matrix consists of temporal correlations in which a row of values represent the synchrony between one network and all the other networks. For a brain FC study, we may cluster the highly correlated components into subgroups (clusters) by applying a functional parcellation procedure to the FC matrix (Venkataraman et al., 2009), producing on-diagonal blocks in the clustered FC matrix. In this work, we did not perform functional clustering on the FC matrix. Instead, we calculated an FC matrix based on the ICA timecourse labels in a descending order of task correlation. We can then straightforwardly analyze the structure and pattern in the task-ordered FC matrix. Specifically, we observed both on-diagonal and off-diagonal blocks in the task-ordered FC matrix. An on-diagonal block with high correlation values in the FC matrix represents a functional cluster (a clique or a strongly coupled subgroup) that behaves synchronously (coherently). An off-diagonal block indicates a homogeneity of inter-clique coupling. A small value in an FC matrix implies no synchrony or irrelevance.

We observed an irregular checkerboard pattern in the task-correlation-ordered FC matrices (see Supplementary Figures S6, S7), which is characteristic of on-diagonal and off-diagonal blocks in different block sizes and shapes, that were randomly distributed with positive and negative values. The off-diagonal blocks assume similar values and homogeneity as the on-diagonal blocks. The irregular mosaic patterns were regenerated across a span of spatial smoothing settings, consistently reproduced through GIG-ICA (Du and Fan, 2013; Du et al., 2016).

Our experimental results revealed that spatial smoothing enlarges the task activation volume measurements for both single-subject and multi-subject ICA decompositions (see Figure 3B). Although the smoothing-caused task blob enlargement may be partially understood from the normal spatial expansion and blurring effect of smoothing, we can explain this more completely in the correlation scale invariance theory (in Equation 7) that accounts for the interaction between spatial smoothing and correlation (Hoeffding, 1994; Liu et al., 2017). Specifically, the spatial smoothing reduces the image noise level and dissimilarity among neighboring voxels, thus increasing the correlations among the neighboring voxels.

Given a FC matrix, we suggest the use of *mean*(FC) for the balance of positive and negative connections, and *std*(FC) for the connection strength (Chen et al., 2017b). Due to cancellation of positive and negative correlations, a small *mean*(FC) value indicates a positive and negative balance in contingence with a large *std*(FC) value. Our experimental results in Figure 4 show that the FC balance [with a minimal *mean*(FC)] was reached in the spatial smoothing with a 2~3-voxel FWHM for the single-subject ICA FC study. This finding also suggests a 2~3-voxel FWHM spatial smoothing.

Our results further show that spatial smoothing enhances the function connection strength [in terms of *std*(FC)] in Figure 4. We can also explain this phenomenon in the correlation scale invariance theory to a considerable degree. The temporal behaviors of the active voxels in an ICA component become more similar due to spatial smoothing and thus increase the temporal correlations (extending the swings of positive and negative correlations in a statistical distribution of FC values). There remains an incomplete understanding of how spatial smoothing changes the FC matrix via ICA. Clearly, the spatial smoothing changes noisy structures of the raw images, which in turn causes a variation in ICA decomposition and thereby produces a different FC matrix. The theoretical study on the interactions among spatial smoothing, ICA, FC (temporal correlation), and group average is an ongoing research topic.

With the same task correlation ordering (for FC study) of ICA components, we calculated *scorr* matrices (in Equation 6) to observe the spatial overlapping among the ICA maps. Our experiments show that spatial smoothing has no obvious effect on the spatial overlap among ICA components (*scorr* < 0.2 in Figure 5 and Supplementary Figures S8, S9).

## Conclusion

Spatial smoothing is a typical preprocessing step for fMRI. Based on single-subject and multi-subject experiments of task fMRI (finger tapping action), we show that the spatial smoothing of raw fMRI data has an effect on ICA-based brain function analysis: a large FWHM smoothing reduces the task extraction performance and enlarges the task activation volume. We also show that the spatial smoothing can increase the functional coupling strengths (in terms of *std*(FC)) while slightly enhancing the positive and negative imbalance, and the spatial smoothing imposes more effect on single-subject ICA FC than on the multi-subject ICA FC. Our experimental results overall show that the spatial smoothing with a 2~3-voxel FWHM achieved an optimal functional connectivity balance [a state with a minimal *mean*(FC)].

## Author contributions

ZC developed the approach, collected the task fMRI data, performed data analysis and drafted the manuscript. VC participated in data collection and data analysis, edited the manuscript and supervised the research.

### Conflict of interest statement

The authors declare that the research was conducted in the absence of any commercial or financial relationships that could be construed as a potential conflict of interest.
