# Supplementary material for: Effect of Spatial Smoothing on Task fMRI ICA and Functional Connectivity
Source: Front Neurosci. 2018 Feb 2;12:15. doi: 10.3389/fnins.2018.00015 (PMC5801305; doi:10.3389/fnins.2018.00015)

Fig. s1 Effect of ICA-based task timecourse extraction (single-subject ICA)

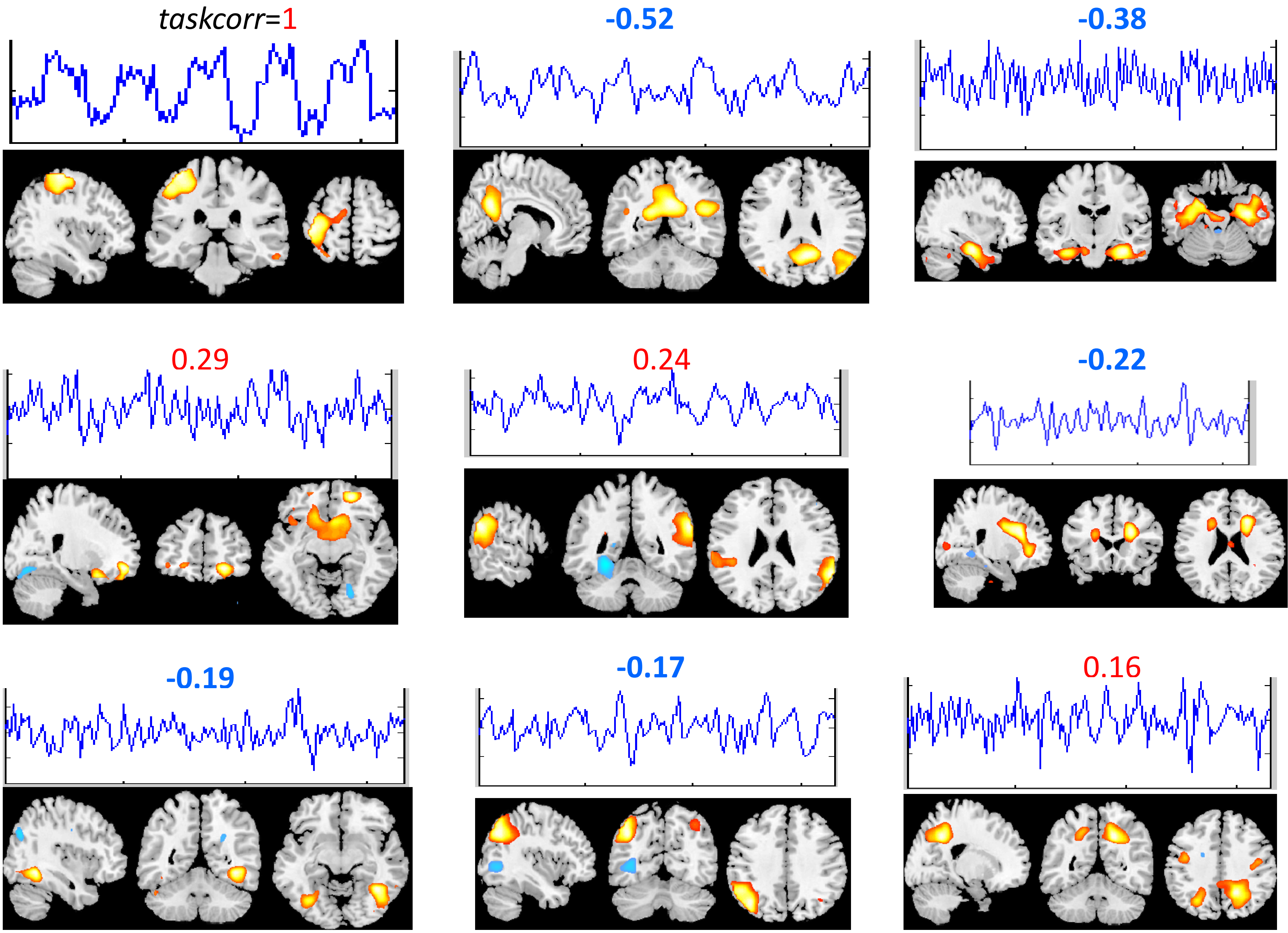

Fig. s2 Effect of ICA-based task timecourse extraction (single-subject ICA)

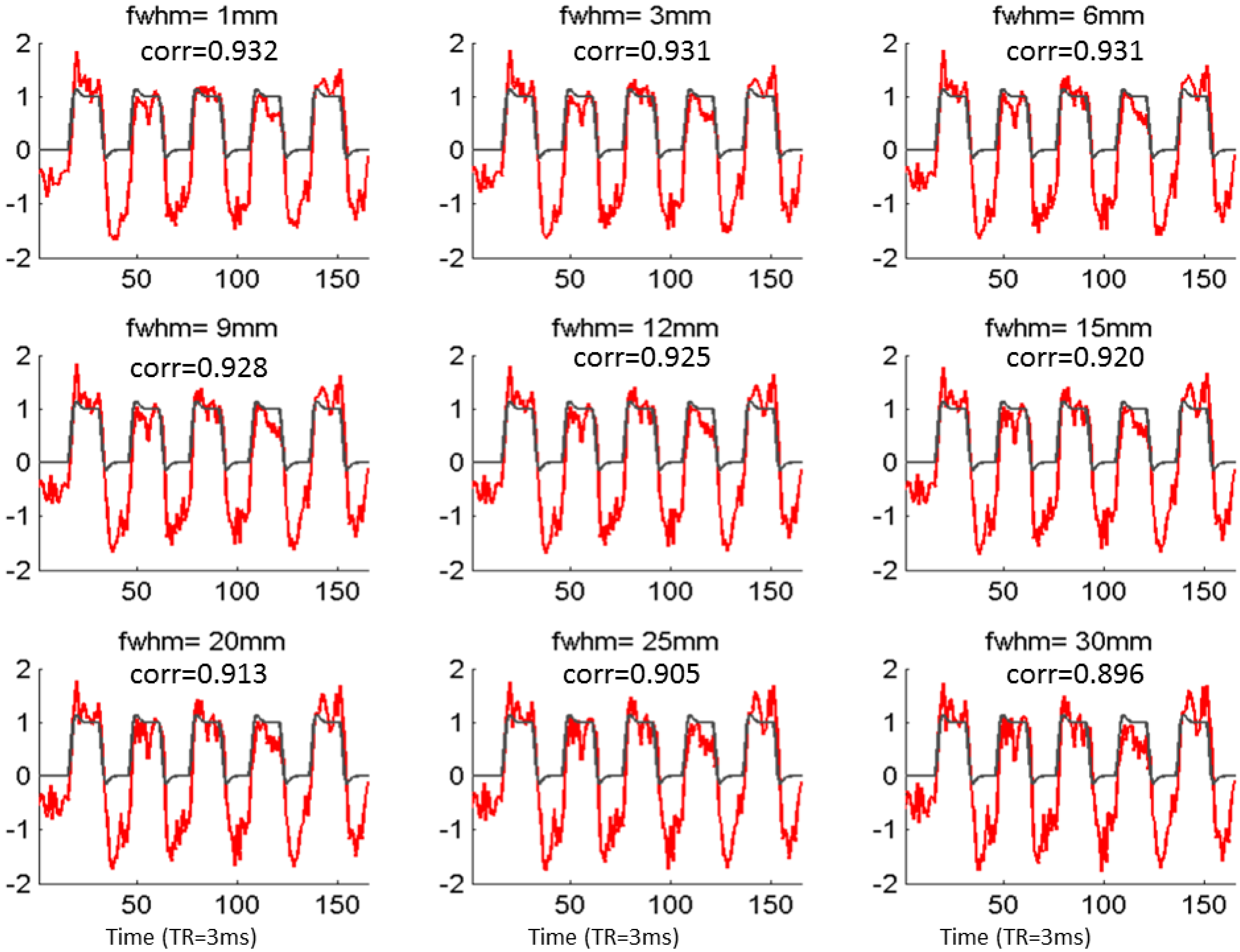

Fig. s3 Effect of ICA-based task timecourse extraction (multi-subject ICA)

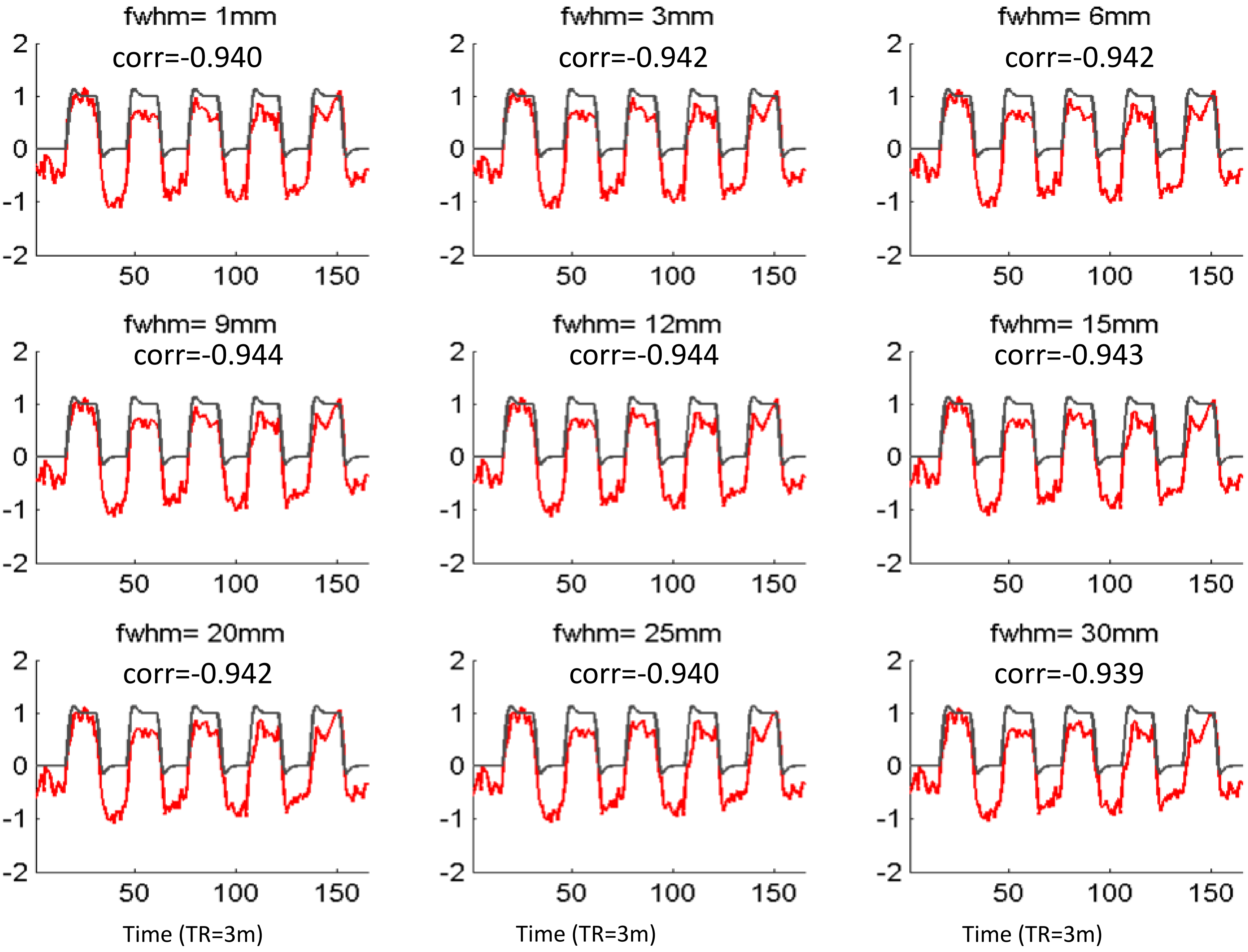

Fig. s4 ICA-extracted task maps from different spatial smoothing (single-subject ICA)

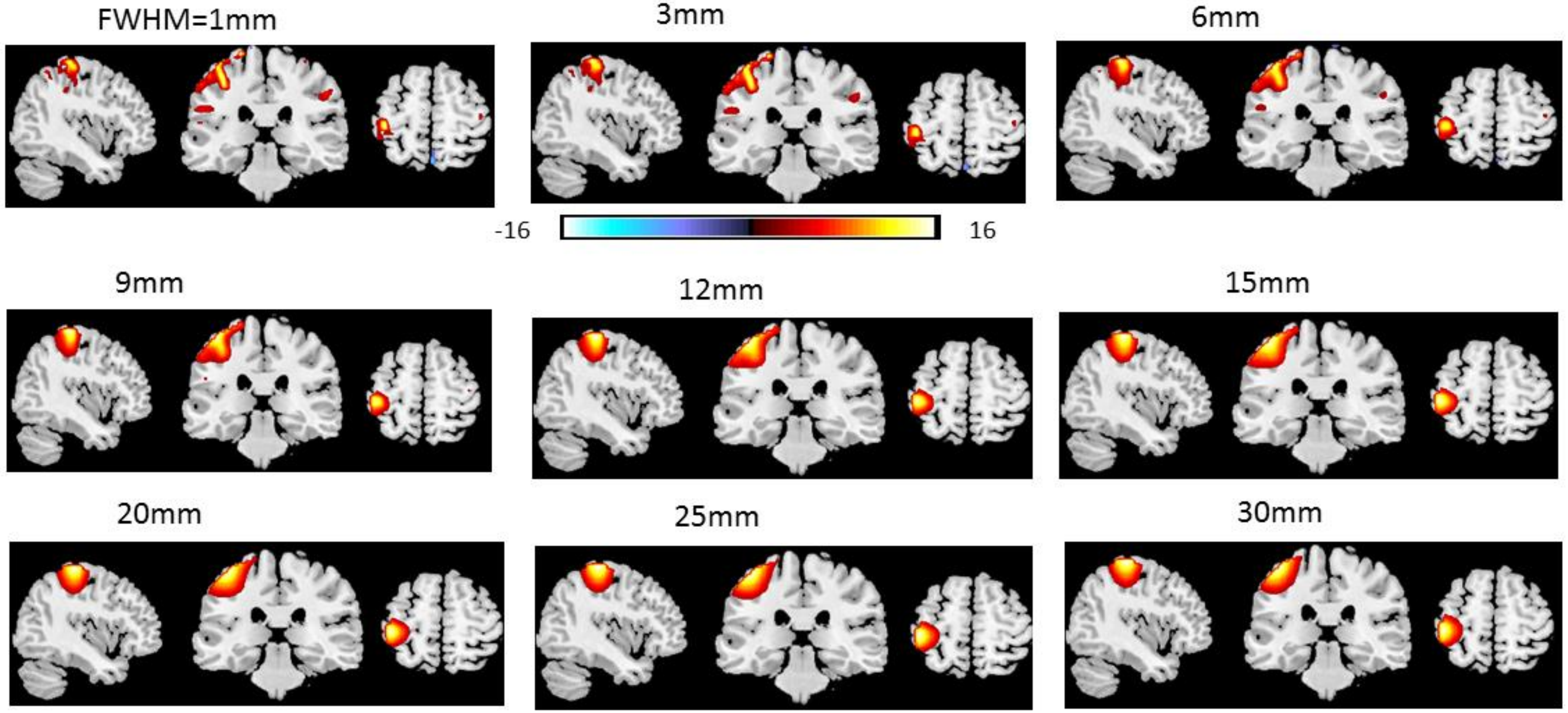

Fig. s5 ICA-extracted task maps from different spatial smoothing (multi-subject ICA)

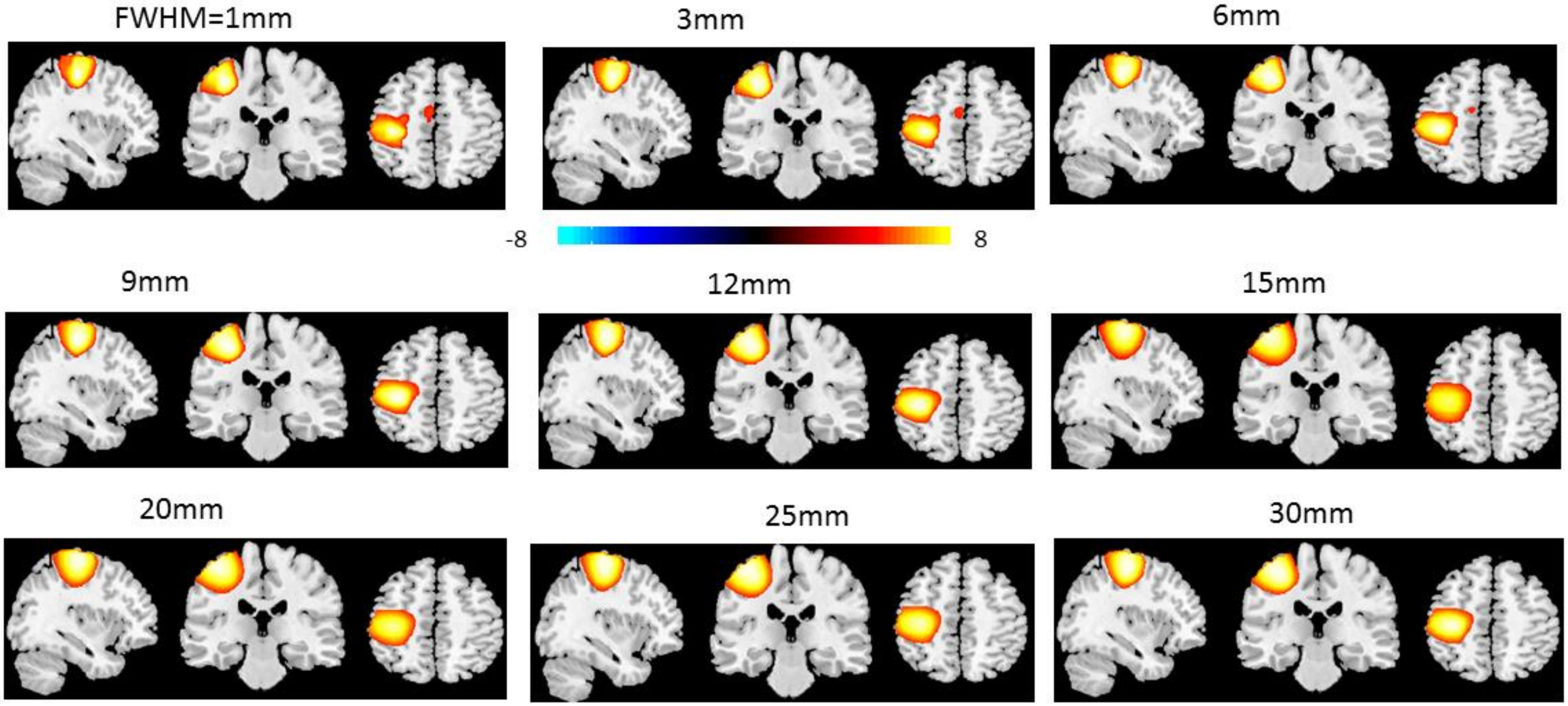

Fig. s6 Effect of spatial smoothing on ICA-based FC matrices (single-subject ICA)

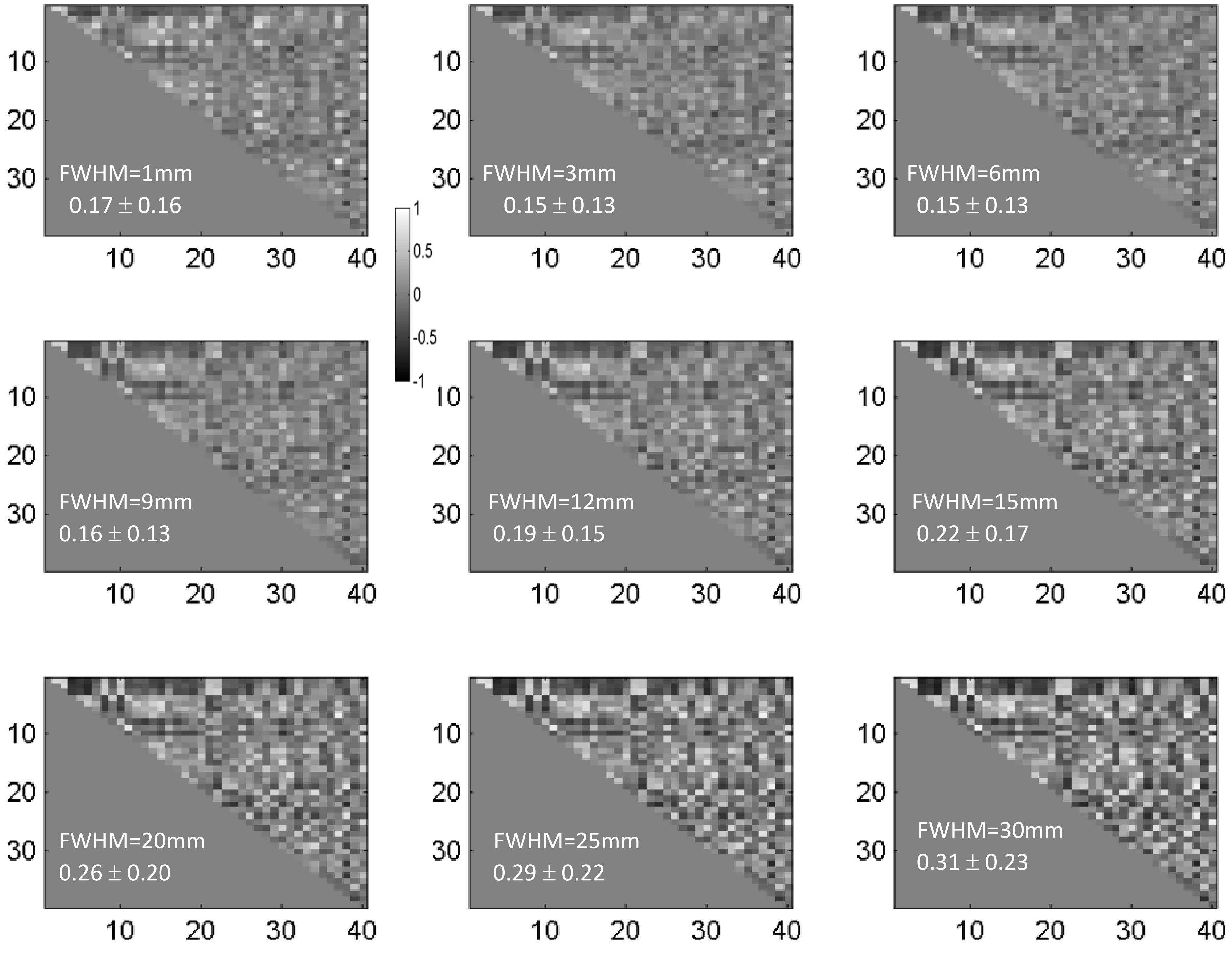

Fig. s7 Effect of spatial smoothing on ICA-based FC matrices (multi-subject ICA)

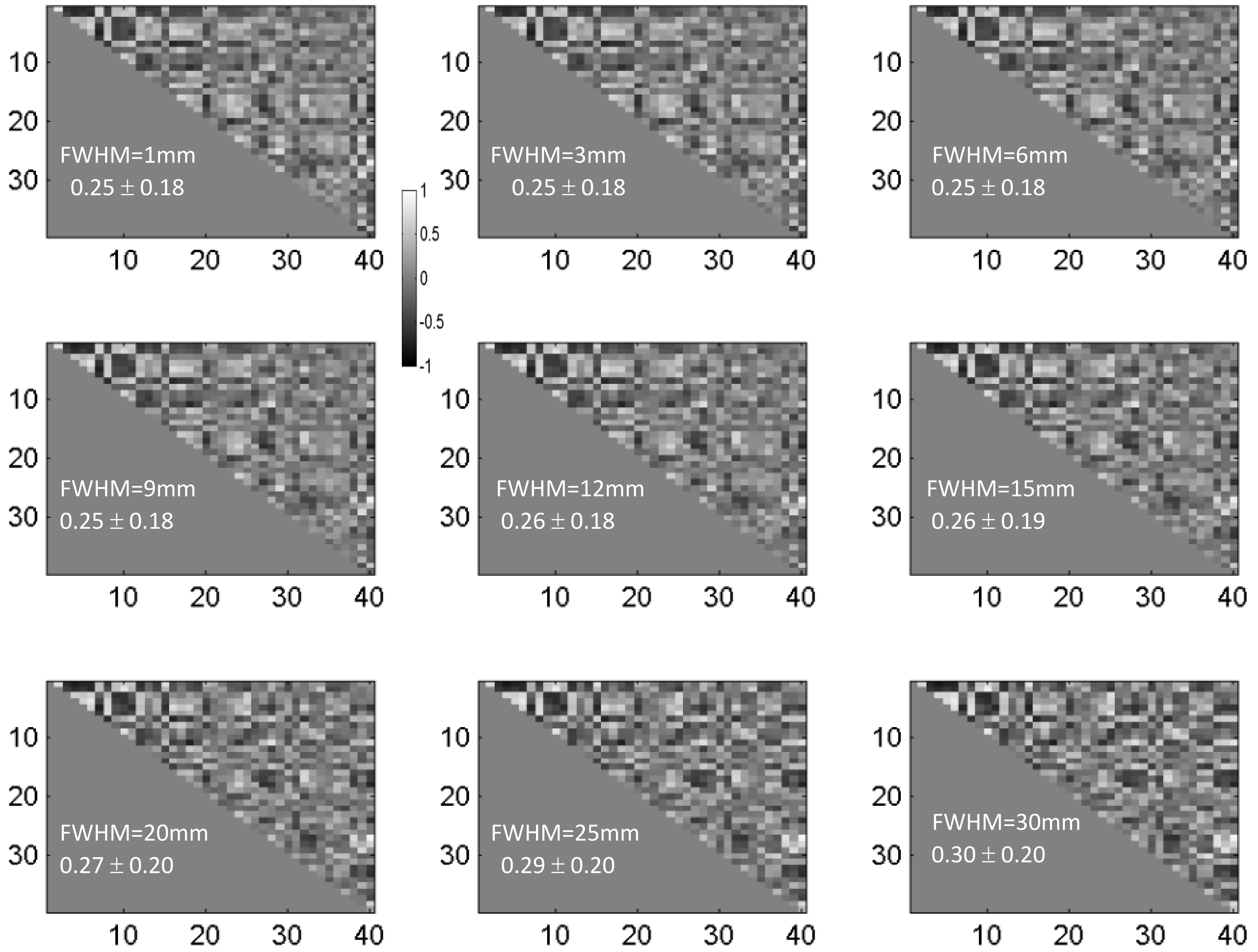

Fig. s8 Effect of spatial smoothing on single-subject ICA component overlapping

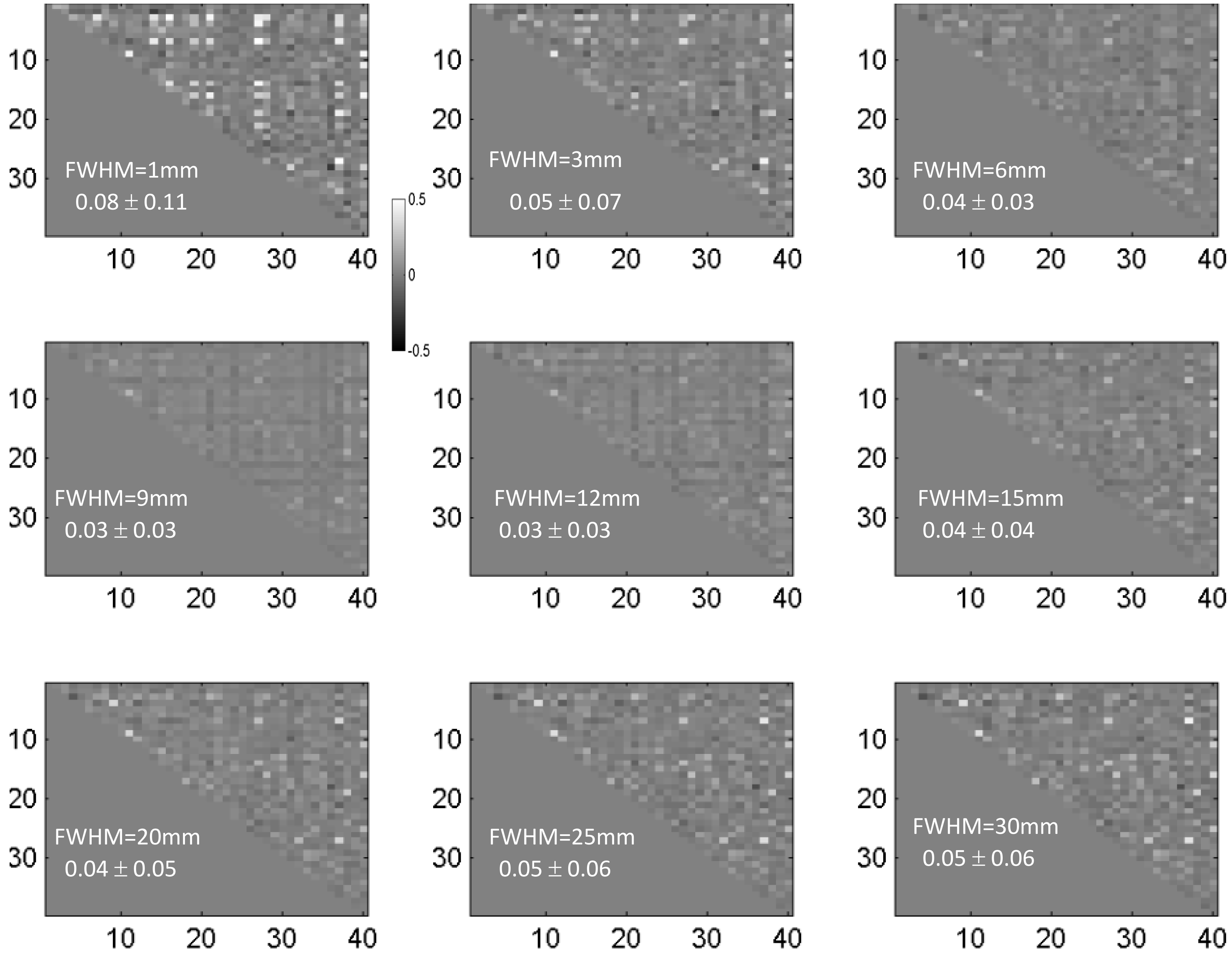

Fig. s9 Effect of spatial smoothing on multi-subject ICA component overlapping

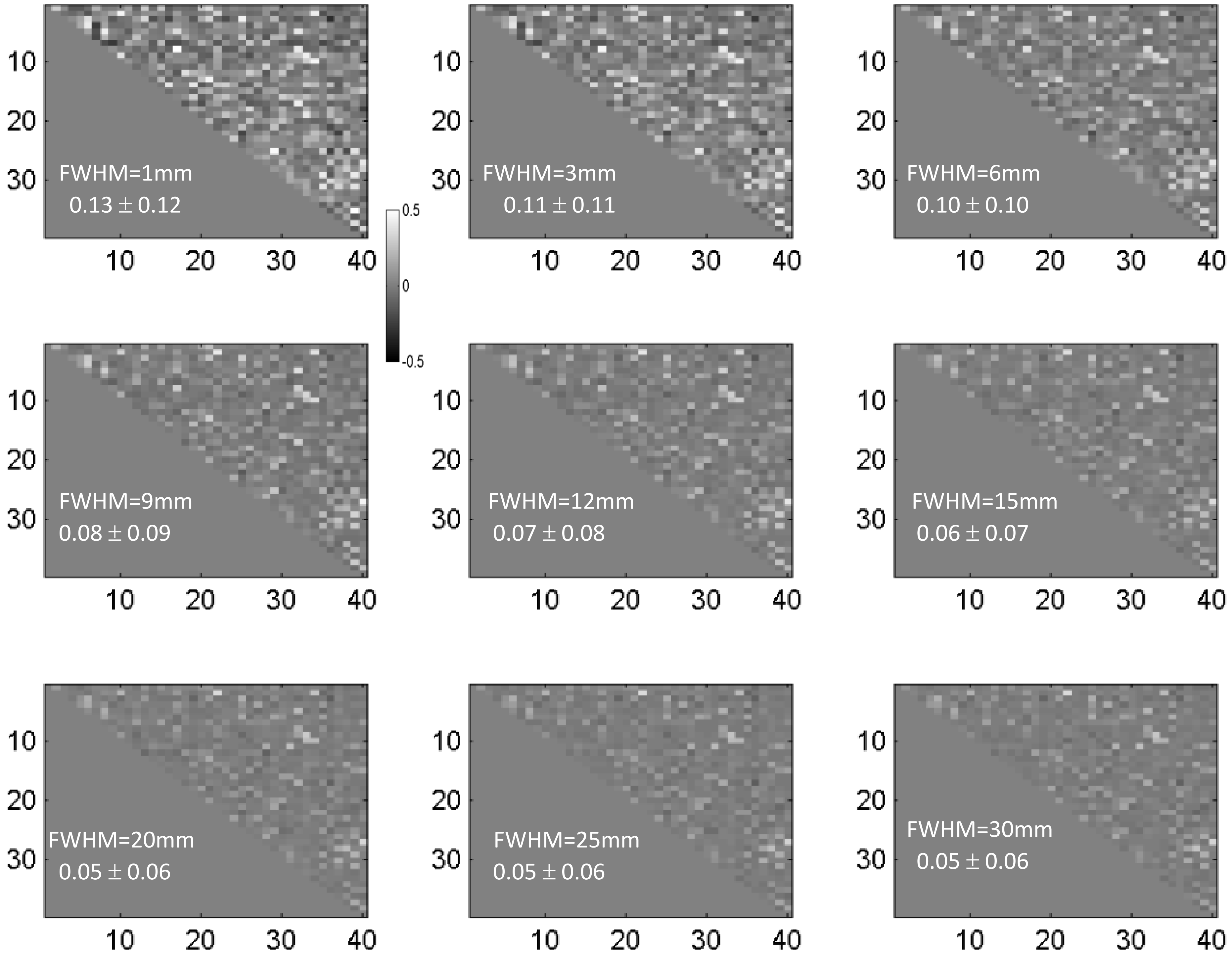

Supplement: Supplementary file 1 [file DataSheet1.pdf]
